# The First Biological Respect Protocol: A Biodigital Technique for Definitive Customized One-Time Abutments—A Case Report

**DOI:** 10.3390/jcm14134448

**Published:** 2025-06-23

**Authors:** Franco Rizzuto, Silvia Rizzuto

**Affiliations:** Private Practice Centro Odontoiatrico Rizzuto, 87100 Cosenza, Italy; silviarizz99@gmail.com

**Keywords:** immediate dental implant, one abutment one time, digital dentistry workflow, customized definitive abutment, guided implant surgery, immediate implant restoration, peri-implant tissues

## Abstract

**Background/Objectives:** Dental implants represent a viable solution for replacing missing teeth; however, multiple disconnections and reconnections of intermediate abutments contribute to the apical displacement of the peri-implant connective tissue barrier, resulting in additional marginal bone loss. To the best of our knowledge, no definitive customized abutments currently exist that are specifically designed according to the morphology of the tooth to be replaced and its position within the dental arch, allowing for digital planning within the prosthetic implant design and insertion during the surgical procedure without subsequent disconnection. **Methods:** The First Biological Respect (FR) technique, described in this case report, enables the digital planning not only of the implant but also of the patented FR customized-shaped, definitive abutment and associated FR prosthetic components. The FR technique was applied to a case involving an immediate post-extraction implant in position 12. **Results**: With the limitations of a case report, the application of the FR protocol demonstrated stable crestal bone levels at the 1-year follow-up. Additionally, soft tissue volume was maintained at 6 months, reflecting the accuracy of the customized prosthetic components in supporting, guiding, and protecting peri-implant soft tissues. At the 1-year follow-up, an increase in soft tissue volume was observed, likely attributable to tissue maturation and the further customization of the definitive prosthetic elements. **Conclusions**: The FR technique represents a viable therapeutic alternative that, through its patented, fully customized components, allows for the digital planning of the implant, as well as the customized definitive abutment, coping, provisional, and final prosthetic framework. This facilitates a single-stage surgical and prosthetic approach. By eliminating the need for repeated abutment disconnections, this method supports the long-term stability of both hard and soft peri-implant tissues while also reducing overall treatment time for both clinician and patient. Further studies involving larger patient cohorts are necessary to validate this protocol.

## 1. Introduction

Implant placement today aims to restore the patient’s function and esthetics in the shortest possible time while considering long-term anatomical, surgical, and prosthetic factors associated with various procedures [1,2,3]. Proper three-dimensional implant placement is determined by the anticipated position of the future prosthetic restoration [4]. In this regard, static or dynamic guided surgery and digital planning allow for increased predictability, safety, and efficiency in implant placement based on the final prosthetic restoration [5,6,7,8]. A key consideration in implant rehabilitation is the stability of peri-implant tissues, particularly in relation to the interactions between the abutment and the implant itself. The manipulation of intermediate abutments during the restorative process can affect peri-implant tissue health, potentially leading to marginal bone loss in order to re-establish the correct mucosal seal [9,10,11,12]. In particular, each disconnection event can result in a marginal bone loss of approximately 0.15–0.2 mm, and repeated abutment manipulations have been associated with a progressive increase in bone loss over time, with cumulative disconnections leading to even greater bone loss [13,14,15,16]. This is due to the disruption of the soft tissue seal and the subsequent apical migration of the junctional epithelium, resulting in the re-establishment of the biological width and marginal bone remodeling [9,10,11,12]. The biological width, a key dimension of the peri-implant mucosa, is re-established after each manipulation of the abutment, underscoring the critical importance of maintaining a stable biological seal around the implant–abutment interface [10,11]. Various strategies have been proposed to minimize these adverse effects, focusing on abutment designs and connections that maintain the biological integrity of the peri-implant tissues. To address these concerns, the concept of “one abutment, one time” has gained attention. This approach involves placing a definitive abutment at the time of implant insertion and avoiding its subsequent removal, thereby preventing disruption of the peri-implant soft tissue seal and maintaining the integrity of the biological width [13,14,15,16,17,18,19]. Studies have demonstrated that this protocol can reduce marginal bone loss by up to 0.5 mm over three years compared to repeated abutment manipulation [15]. For example, Canullo et al. and Degidi et al. reported significantly less crestal bone loss in implants following the one abutment–one time approach versus those subject to multiple abutment changes [13,14]. Tallarico et al. further confirmed in a systematic review that definitive abutments placed and never removed result in a reduction in mean marginal bone loss by approximately 0.41 mm after one year [16]. These findings emphasize that preserving the soft tissue seal and reducing the frequency of abutment disconnections are critical for long-term peri-implant tissue health. Biologically, this approach supports the natural processes of osseointegration and soft tissue integration around dental implants. Osseointegration establishes a direct structural and functional connection between bone and the implant surface, ensuring primary stability [1,2,3]. Similarly, the establishment of an effective soft tissue seal around the implant–abutment interface serves as a critical barrier, protecting the underlying bone from bacterial infiltration and mechanical trauma [9,10,11]. Repeated disconnections can compromise this barrier, leading to chronic inflammatory responses and bone remodeling [12]. Therefore, the adoption of the one abutment–one time protocol aligns with the biological principles of implant success, preserving both the bone and soft tissue architecture [15,16,17]. Regarding soft tissue stability and esthetics, the placement of an immediate provisional prosthetic restoration, particularly in immediate implants, supports tissue architecture and ensures healthy peri-implant conditions by maintaining the mucosal seal. This is further supported by the precision fit of implant–abutment and abutment–restoration connections [20,21,22,23,24,25]. To the best of our knowledge, as of today, one-time abutments are round in shape and not specifically customized based on the tooth to be replaced or the arch in which they are located. Moreover, according to the literature, customized abutments are typically healing abutments or are fabricated using indirect techniques that do not eliminate intermediate disconnections and impressions. The ‘First Biological Respect’ (FR) technique described in this paper refers to a complete digital method encompassing diagnosis, design, and execution. In addition to the three-dimensional planning of implant placement, the patented customized definitive abutment, tailored to the specific tooth being replaced and its position in the arch, is incorporated into the implant–prosthetic design. The customized coping, provisional restoration, and definitive restoration framework, designed on the customized definitive abutments, are prepared in advance and available in the clinic prior to surgical implant placement, enabling complete biodigital restorations. By placing the customized definitive abutment and other individualized prosthetic components at the time of implant insertion, the FR technique adheres to the “one abutment–one time” concept, preserving the soft tissue seal and minimizing marginal bone loss. However, unlike traditional one-time, round-shaped abutments, FR abutments are digitally customized using mathematical models to precisely conform to the unique anatomy of the tooth being replaced and its specific position within the dental arch. This case report outlines the FR protocol as a therapeutic alternative that supports biological healing mechanisms by stabilizing the blood clot, promoting soft tissue maturation, and maintaining the integrity of the bone-to-implant interface, while also reducing treatment time, the need for impressions, intermediate steps, disconnections, and both biological and economic costs.

## 2. Case Presentation

**Figure 1:**
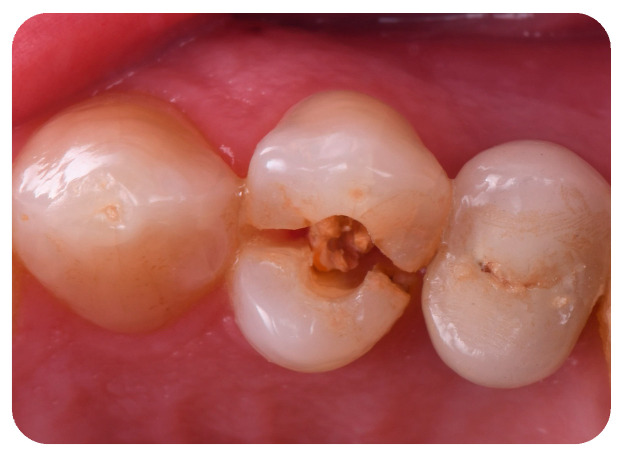
Intraoral initial situation of the left maxillary first premolar.

A 60-year-old female patient, in good health and a non-smoker, presented to our clinic in September 2022 with a fracture of the left maxillary first premolar (Figure 1). The case required replacement of the fractured tooth and placement of a definitive restoration with minimal chairside and waiting time. Diagnostic data were collected using cone beam computed tomography (CBCT), digital impression, and photographs. Of the various treatment options proposed, the patient opted for immediate implant placement with immediate restoration using the “First Biological Respect” (FR) method, intended to replace the left maxillary first premolar. Clinical parameters, as well as peri-implant hard and soft tissue assessments, were evaluated at 6-month and 1-year follow-up.

### 2.1. Clinical Procedures

#### 2.1.1. Phase 1: Digital Planning of the Implant, Customized Definitive Abutment, Coping, Provisional, and Definitive Framework

**Figure 2:**
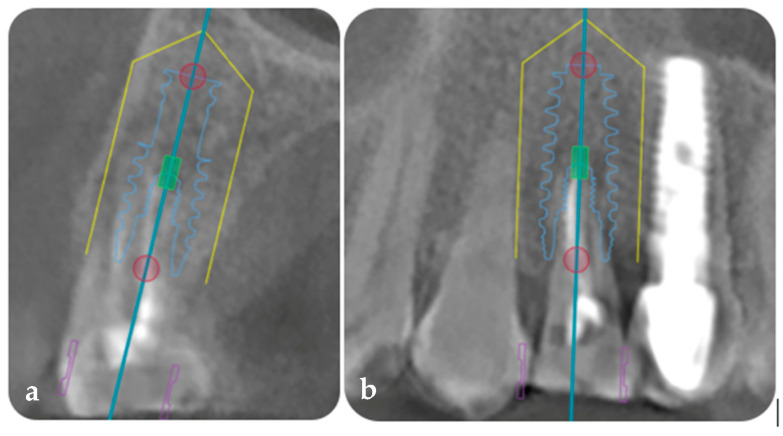
(**a**) Three-dimensional implant planning in the sagittal plane using paired DICOM and STL files. (**b**) Three-dimensional implant planning in the frontal plane using paired DICOM and STL files. The yellow color represents the safety distance limit between the implant and adjacent elements, the blue represents the axis of the implant and its outline.

DICOM and STL files were exported to an implant planning software (Exoplan Studio 3.0, GmbH, Darmstadt, Germany) to plan the prosthetically guided implant placement in three dimensions. A Biomet 5/4 × 13 mm tapered implant with an internal connection was planned, taking into consideration the digital design for the correct mesio-distal positioning of the implant to be ≥1.5 mm from the adjacent natural tooth and ≥3 mm between implants, as well as the equi-crestal position in the coronal–apical direction (Figure 2a,b).

**Figure 3:**
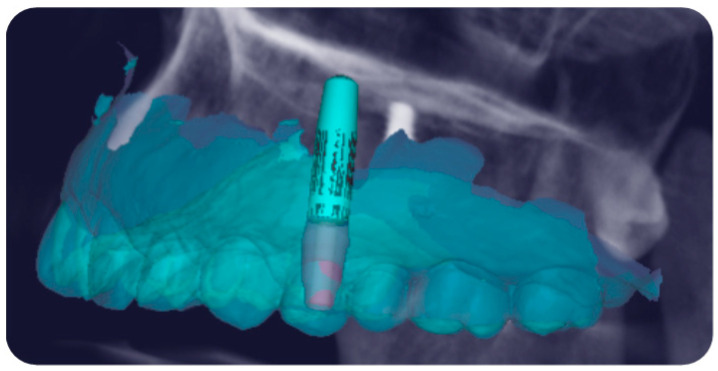
Placement in digital design of the definitive FR abutment on the implant, customized and selected from a patented library according to the tooth to be replaced and the arch in which it is located.

This protocol involves a biodigital workflow that allows for the three-dimensional planning not only of the implant but also of the customized prosthetic components, including the definitive customized abutment. This is made possible by the patented digital mathematics, derived from statistical and mathematical analyses that have enabled the design and production of FR prosthetic components specific to the tooth being replaced and its location within the dental arch, which allow their integration into the implant–prosthetic project. Thus, the customized definitive FR titanium abutment, tailored to the tooth and the arch in which it is located, was designed on the implant (Figure 3).

**Figure 4:**
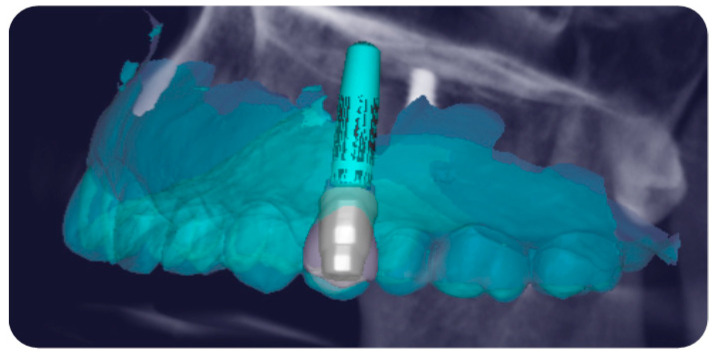
Placement in digital design of the customized FR coping, provisional and definitive framework on the selected FR abutment.

The design of the implant and FR abutment was then transferred to prosthetic software (Exocad 3.0, GmbH, Darmstadt, Germany) to facilitate the placement of the customized FR coping, provisional restoration, and definitive prosthetic framework, on which the esthetics would be finalized (Figure 4).

The definitive abutment and all customized prosthetic components were directly produced from the digital plan and were available in the clinic prior to implant surgery, thereby reducing several intermediate steps. With the FR technique, to ensure accuracy and precision in implant placement and prosthetic component positioning, it is preferable to perform the procedures using guided surgery. If the anatomical site does not permit static guided surgery with a surgical template, dynamic guided surgery can be performed instead.

#### 2.1.2. Phase 2: Implant Placement, Delivery of the Definitive Customized Abutment, Coping, Provisional Restoration, and Fitting of the Definitive Framework

**Figure 5:**
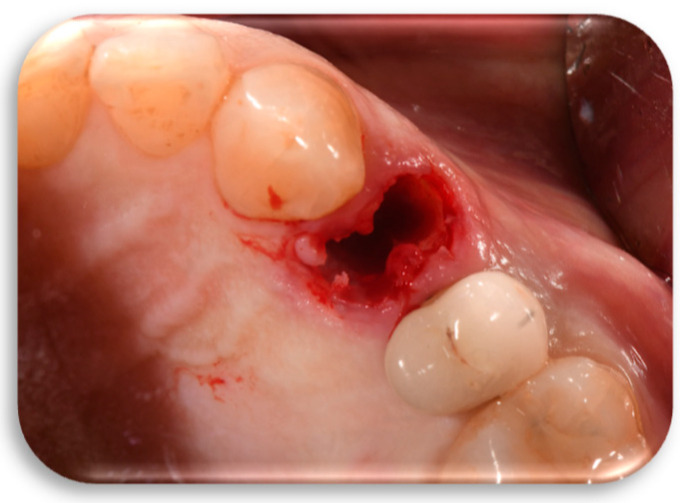
Minimum traumatic tooth 12 (ADA Universal Tooth Designation System) extraction and post-extraction cavity.

Flapless extraction of the tooth in position 12 (ADA Universal Tooth Designation System) with a thick phenotype was performed in a minimally traumatic manner (Figure 5).

The selected implant (Biomet, 5/4 × 13 mm tapered implant with internal connection) was placed according to the prosthetic surgical design using dynamic guided surgery, respecting the proper distance between the adjacent mesial tooth and distal implant (X Guide, X-Nav Technologies, Lansdale, PA, USA). The dynamic navigation protocol was adopted to enable real-time, computer-assisted surgery, allowing the clinician to precisely control implant depth, angulation, and position during the procedure. By using optical tracking systems connected to both the patient and the surgical handpiece, continuous feedback was ensured, resulting in high accuracy [5,6,7,8].

**Figure 6:**
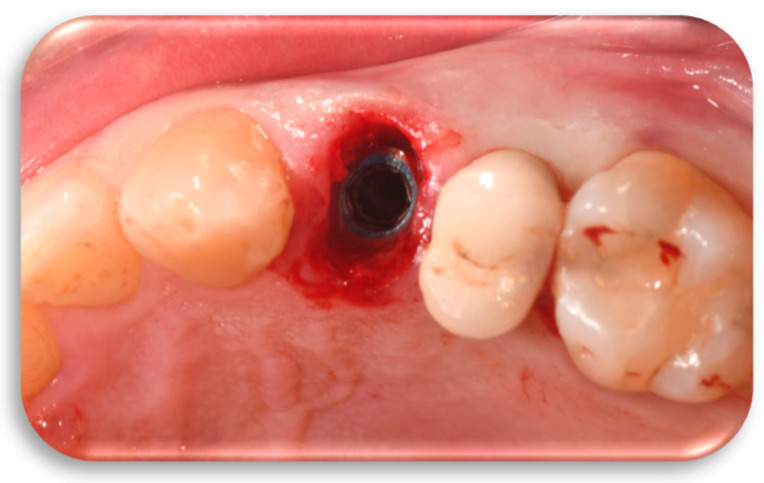
Dynamic guided surgery Biomet 5/4 × 13 mm implant placement according to the surgical–prosthetic design.

The implant was inserted with a torque of 50 newtons (N), and one side of the internal hexagon of the implant was positioned in the vestibular direction using manual torque. An implant stability quotient (ISQ) > 60 was recorded through the resonance frequency analysis system (Penguin, Integration Diagnostics Ltd., Goteborgsvagen, Sweden) (Figure 6).

**Figure 7:**
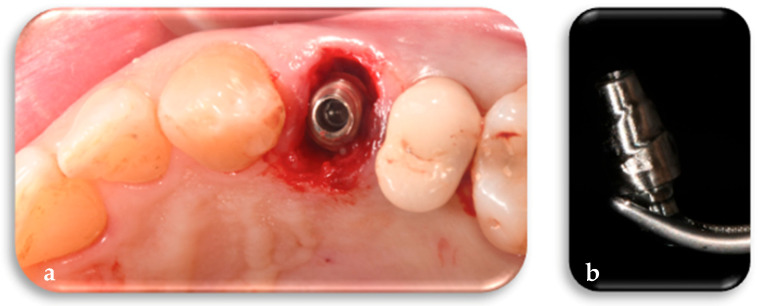
(**a**) Positioning of the customized definitive FR abutment, based on the tooth to be replaced, on the implant according to the digital design. (**b**) Detail of the FR abutment, specifically designed for the left first premolar, produced from the digital design and integrated with the other FR prosthetic components.

The titanium definitive FR abutment has a customized profile in its mesial, distal, buccal, lingual, and height dimensions as well as inclinations, depending on the tooth to be replaced and the arch in which it is located. The FR abutment was placed and screwed onto the implant with a torque of 25 N, ensuring alignment between the internal hexagon of the implant, previously positioned with one of its sides in the vestibular direction, and the internal hexagon of the definitive abutment. This alignment allowed the vestibular face of the FR abutment to correspond with the side of the internal hexagon of the implant, thereby preventing rotation and loosening (Figure 7a,b).

**Figure 8:**
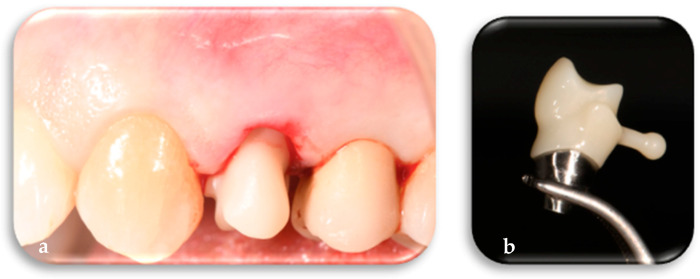
(**a**) Trial of the definitive framework on the FR abutment according to the digital design. (**b**) Detail of the final FR framework, specifically designed for the left first premolar, produced from the digital design and integrated with the other FR prosthetic components.

The definitive framework is digitally designed on the customized definitive abutment and fabricated prior to surgery along with the other FR components. Following placement of the FR abutment on the implant, the definitive framework is intraoperatively tested using a conometric connection to verify its precision and fit. Subsequently, the framework is temporarily set aside and will be utilized to finalize the definitive restoration once peri-implant tissue maturation has occurred (Figure 8a,b).

**Figure 9:**
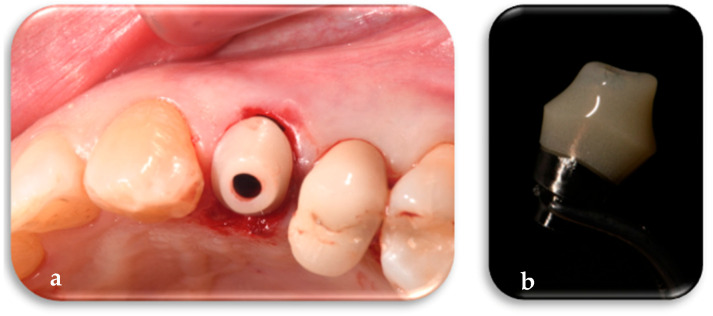
(**a**) Conometric positioning of the customized FR coping on the implant according to digital design. (**b**) Detail of the FR coping, specific for the left first premolar, produced from the digital design and integrated with the other FR prosthetic components.

The customized polymethyl methacrylate (PMMA) coping, shaped specifically according to the tooth to be replaced and the corresponding dental arch, contributes to the customization of the most coronal part of the osseomucosal seal. The specific FR coping was placed conometrically onto the corresponding FR abutment. The conometric connection is a mechanical retention system based on frictional coupling between complementary conical surfaces, allowing a stable fit free of micromovements between the FR coping and the FR abutment. This system ensures effective bacterial seal and clinical retrievability without the use of screws or cement (Figure 9a,b).

**Figure 10:**
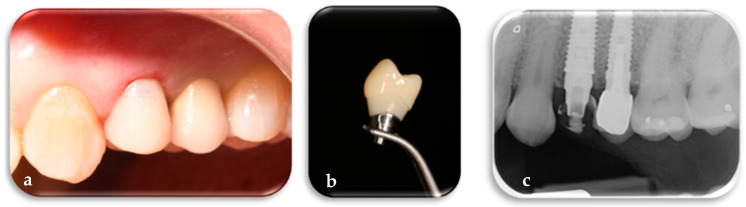
(**a**) Conometric provisional restoration placed on the FR coping according to the digital design. (**b**) Detail of the provisional restoration created from the digital plan, showing integration with other FR prosthetic components. (**c**) Periapical radiograph of the initial situation, illustrating all prosthetic components in place.

The provisional restoration, also made of PMMA, was conometrically placed on the coping and positioned out of occlusion to minimize micromovements at the bone–implant interface during the early healing phase, thereby reducing the risk of implant instability and promoting successful osseointegration (Figure 10a) [22,23,24,25]. Clinical photographs were taken during and immediately after surgery. A periapical radiograph, using a custom-made bite block, was taken immediately after surgery (Figure 10b,c). The patient was prescribed anti-inflammatory medication (Ibuprofen 600 mg every 8 h as needed) and instructed to rinse with 15 mL of 0.12% chlorhexidine for 7–10 days.

#### 2.1.3. Phase 3: Finalization of Aesthetics on the Definitive Framework and Follow-Up

**Figure 11:**
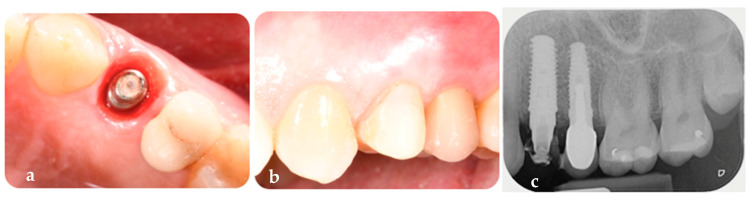
(**a**) Peri-implant osseomucosal seal achieved at 6-month follow-up with the definitive and customized FR abutment, FR coping, and provisional restoration. (**b**) Follow-up at 6 months in vestibular view with the provisional restoration in place. (**c**) Periapical radiograph at 6 months after implant placement, showing the FR abutment, FR coping, and provisional restoration.

Once the peri-implant tissues had matured, typically after 3 to 6 months according to the literature, the final framework, which had been digitally designed on the definitive abutment and tested on the day of surgery, was placed on the FR abutment to take a mucostatic digital impression [17,26,27,28,29]. This impression was used to record the tissues in their resting state, ensuring that the natural form and contours were captured without distortion. The mucostatic impression allowed for the accurate recording of the mature soft tissue profile, enabling the finalization of the esthetics on the previously produced final framework. This ensured that the final prosthetic restoration conformed to the correct morphology of the mature peri-implant tissues. The clinical situation at 6 months was documented using standardized photographs, digital scans, periapical radiographs using a custom-made bite block, and clinical measurements (Figure 11a–c).

**Figure 12:**
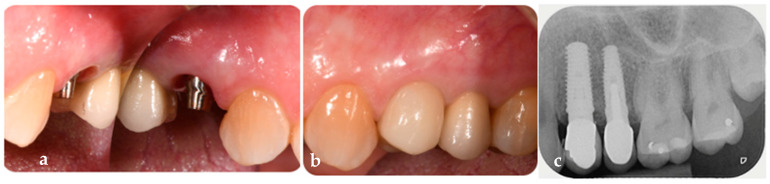
(**a**) Profile of the peri-implant osseomucosal seal before the placement of the definitive crown. (**b**) Delivery of the definitive prosthetic crown in vestibular view. (**c**) Periapical radiograph at 1-year follow-up with the definitive crown in place.

The definitive crown, made of layered zirconia, was placed conometrically on the FR customized definitive titanium abutment without the use of cement or screws. The abutment has never been removed since its initial placement during the surgery. At the 1-year follow-up, the final crown demonstrated stability and health of the peri-implant tissues. Follow-up evaluations, including periapical radiograph using a custom-made bite block, digital scan, clinical measurements, and standardized photographs, were conducted (Figure 12a–c).

### 2.2. Measurements

**Figure 13:**
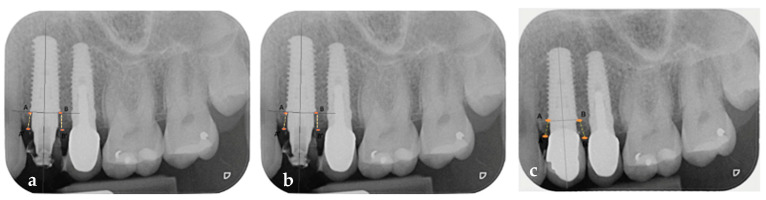
(**a**) Mesial and distal bone peak measurement on periapical radiograph taken at T1. (**b**) Mesial and distal bone peak measurement on periapical radiograph taken at T2. (**c**) Mesial and distal bone peak measurement on periapical radiograph taken at T3. A-A′ represents the measurement of the mesial bone peak to the implant, defined as the vertical distance between the most coronal point of the mesial alveolar bone adjacent to the implant and the line passing through the head of the implant. B-B′ represents the measurement of the distal bone peak to the implant, defined as the vertical distance between the most coronal point of the distal alveolar bone adjacent to the implant and the line passing through the head of the implant.

Periapical radiographs taken at the time of the placement of the implant, customized definitive abutment, coping, and provisional restoration (T1) (Figure 13a), after 6 months (T2) (Figure 13b), and at 1 year (T3) (Figure 13c) were compared to assess the trend in the mesial and distal bone peaks.

**Figure 14:**
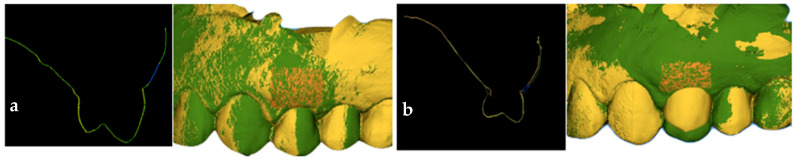
(**a**) Evaluation of volumetric changes by superimposing digital scans taken at T0 and T2. The yellow color represents the scan performed at T0, the green color represents the scan performed at T2, the orange color indicates the rectangular area considered for calculating the volumetric change, and the blue color represents the volume variation. (**b**) Evaluation of volumetric changes by superimposing digital scans taken at T2 and T3. The yellow color represents the scan performed at T2, the green color represents the scan performed at T3, the orange color indicates the rectangular area considered for calculating the volumetric change, and the blue color represents the volume variation.

The three periapical radiographs were calibrated to a sensor size of 31 mm × 41 mm to ensure repeatability and comparability of measurements. In the three radiographs, the line passing through the head of the implant was identified, and the distance between this line and the mesial and distal bone peaks was measured [30] (ImageJ software 1.54, National Institutes of Health, Bethesda, MD, USA). The mesial bone peak value of 2.8 mm did not change significantly from T1 to T3, and the distal bone peak value of 2.67 mm also did not change significantly from T1 to T3. Digital scans taken before the placement of the implant, customized definitive abutment, coping, and temporary restoration (T0); at 6 months after their placement (T2); and at 1 year (T3) were superimposed to observe changes in soft tissues. From the superimposition of the T0–T2 digital scans, a rectangular area defined apico-coronally by the gingival margin of the tooth and the muco-gingival line, and mesio-distally by a vertical line passing through the center of the interproximal papilla, was selected (SMOP Swissmeda Software 2.21.4, Montagnola, Svizzera). A volumetric increase of 0.57 mm^3^ was recorded (Figure 14a) [31]. From the superimposition of the T2–T3 digital scans, the same rectangular area of interest was selected, and a volumetric increment of 22 mm^3^ was recorded (Figure 14b).

Clinical measurements were recorded at the 6-month and 1-year follow-ups at six sites per implant using a periodontal probe (PCP UNC-15, Hu-Friedy, Chicago, IL, USA). The following outcomes were assessed: implant, abutment, or prosthesis mobility; presence or absence of keratinized peri-implant mucosa in the mid-buccal and mid-lingual aspects; sulcus bleeding index (SBI); modified plaque index (mPLI); and probing depth (PD) [26]. Keratinized mucosa width (KMW) was measured using a periodontal probe at the site prior to implant extraction and at 6-month and 1-year follow-ups at the mid-buccal aspect [10].

## 3. Discussion and Limitations

Despite the limitations related to potential distortions of periapical radiographs due to X-ray beam divergence, measurements taken from periapical radiographs indicated that changes in the mesial and distal bone peaks around the implant appeared to be minimal one year after implant placement, with the crestal bone level remaining coronal to the implant platform. These outcomes align with previous evidence indicating that maintaining a stable peri-implant environment minimizes marginal bone loss and soft tissue recession [9,12,13,14,15,16,17,18,32]. The FR definitive customized abutment, by precisely guiding and preserving the clot from the outset, appears to mitigate apical displacement of the peri-implant connective tissue barrier and marginal bone loss. This is crucial, as repeated disconnections and reconnections of intermediate prosthetic components have been widely documented to compromise peri-implant tissue stability [9,12,13,14,15,16,17,18,32].

Clinical measurements further supported this, showing the following:

- Absence of mobility of the prosthesis, abutment, and implant at both 6-month and 1-year follow-up.
- Presence of keratinized peri-implant on the mid-buccal and mid-lingual aspects at 6-month and 1-year follow-up.
- Sulcus Bleeding Index (SBI) score of 0 at both 6-month and 1-year follow-up.
- Modified Plaque Index (mPLI) score of 0 at both 6-month and 1-year follow-up.
- Probing Depth (PD) < 4 mm at both 6-month and 1-year follow-up.
- Keratinized mucosa width (KMW) values of 4 mm before surgery, 4 mm at 6 months, and 5 mm at 1 year after implant, customized abutment, coping, and restoration placement.

These findings are in line with the “one abutment, one time” concept, which is supported by extensive data showing reduced bone loss and improved soft tissue stability compared to multiple disconnections.

Measurements taken from the superimposed STL files demonstrated that, at 6 months following the placement of the implant, the definitive customized abutment, customized coping, and provisional restoration, the soft tissue volume did not change and remained stable. Therefore, the precise morphology of the customized prosthetic components, produced before implant placement and positioned at the time of implant insertion, provided adequate soft tissue support due to the stability and differentiation of the clot. At the 1-year follow-up, an increase in volume was observed, associated with tissue maturation and further customization of the prosthetic components and the final crown, which enhanced tissue support. This outcome is consistent with studies showing that precise emergence profiles and proper tissue support are key to long-term stability [33,34]. The clinical and digital findings of this case report highlight how the definitive, site-specific customization of the FR abutment immediately at the time of implant placement—unlike standard round-shaped one-time abutments—contributes to early peri-implant tissue conditioning and avoids the biological complications of repeated abutment manipulations [27,35,36].

However, additional clinical studies are needed to validate this method and to assess both hard and soft tissue changes over time across different implant placement protocols. Importantly, our observations underscore that digitally guided implant surgery ensures precise three-dimensional positioning of both the implant and its prosthetic components, thereby achieving a prosthetically driven and biologically compatible outcome [1,2,3]. The use of digital design software allows for proper implant and restoration placement, facilitating the identification and management of critical anatomical and biological factors. Guided surgery enables the direct transfer of the virtual plan to the clinical setting with a high degree of precision and accuracy, surpassing the predictability of freehand implant placement. In agreement with the literature, guided navigation systems allow for intraoperative adjustment of planned implant placement and control of drilling in critical anatomical situations with a reported mean deviation of less than ~0.30 mm [5,6,7,8]. Moreover, digital workflows enable pre-operative design and fabrication of all components—implant, customized abutment, coping, and final restoration—thereby streamlining the surgical–prosthetic sequence and supporting biological tissue preservation [12,20,32].

In daily clinical practice, to achieve the final prosthetic restoration of an implant, various disconnections and reconnections of intermediate prosthetic elements and different impressions are performed, attempting to condition the peri-implant soft tissues by achieving the best morphology of the mucosal seal. This is primarily due to the fact that healing abutments typically have a circular cross-sectional profile, necessitating modification of the emergence profile to mimic the natural root anatomy prior to final restoration placement. To facilitate this tissue conditioning, customized healing abutments fabricated through indirect techniques are employed, applying controlled pressure to the peri-implant soft tissues [27,35,36]. However, the pressure applied for soft tissue shaping, combined with the repeated disconnection and reconnection of intermediate prosthetic components, leads to disruption of the peri-implant mucosal seal, resulting in increased transmucosal barrier height, bone remodeling, and mucosal recession [9,12,13,17,27,32,35,36].

Abutment disconnections and reconnections impact peri-implant tissues, as shown by an experimental study in dogs conducted by Abrahamsson and colleagues [9]. This study shows apical migration of the peri-implant connective barrier and subsequent marginal bone loss following disconnection and reconnection of the abutment, which is required to re-establish the correct mucosal seal, as extensively discussed by Berglundh and colleagues and Moon and colleagues [9,10,11]. Additionally, Grandi et al., in a 1-year follow-up of a multicenter randomized controlled trial, confirm that the disconnections and reconnections of provisional versus definitive abutments result in changes in marginal bone levels, with an average difference of 0.48 mm [12]. To mitigate the effects of manipulating temporary abutments on peri-implant tissues, the concept of “one abutment, one time” is introduced, involving the placement of the definitive round-shaped abutment immediately at implant insertion, which, according to the best scientific evidence, maintained peri-implant tissue levels over time [13]. Several studies demonstrate lower values of marginal bone resorption and mucosal recession, which remain stable over time in patients treated with the definitive abutment, as it contributes to peri-implant bone stability compared to the use of multiple abutments [13,14,15,16,17,18].

Comparing the one-time abutment and protocols involving several disconnections and reconnections, greater marginal bone loss was observed in the second group at both 6 months and 1 year after prosthetic loading [26,27,28,32,33,34,35,36]. However, some authors report no significant differences in marginal bone resorption between definitive abutment placement and disconnections and reconnections [29]. Differences in results can be attributed to the different follow-up and surgical–prosthetic protocols applied, for example, the use of platform-switching, which is considered a protective factor compared to platform matching, or different abutment heights [26,37]. Disconnections and reconnections also contribute to deformation of the individual connection components, including the internal implant connection surfaces and screw threads, leading to abutment instability. Furthermore, disconnections are associated with the passage of infected, contaminated fluids, thereby creating an environment conducive to the proliferation of anaerobic bacteria [16,19]. Moreover, to prevent marginal bone loss and enhance peri-implant tissue integrity, the literature suggests that cone-in-cone abutment–restoration connections, which rely on friction to ensure a precise marginal fit, help prevent bacterial infiltration and promote marginal tissue health over time [22,23,24,25]. To date, digital technologies such as CAD/CAM systems enable the fabrication of customized abutments and prosthetic restorations from digital scans. However, this process is not exempt from disconnections of intermediate prosthetic components and multiple impressions and does not allow for the delivery of the definitive customized abutment at the time of implant placement [36,38].

The FR protocol proposed in this case report, to the best of our knowledge:

- Differs from the “one abutment, one time” protocol because FR abutments are not round-shaped but definitive and customized, specifically designed according to the tooth to be replaced and the arch in which it is located.
- Differs from customized healing abutments fabricated using indirect techniques, which are not exempt from disconnections and reconnections, as FR abutments are definitive and digitally customized based on the digital mathematics of the tooth to be replaced and the corresponding arch.
- Allows for digital planning not only of the implant placement but also of the customized prosthetic components—definitive abutment, coping, provisional restoration, and final prosthetic structure—thereby reducing working time, waiting time, the number of disconnections, components, and intermediate impressions.

The FR method enables the placement of the customized definitive abutment, customized coping, temporary, and final framework within the digital design, utilizing patented digital mathematics based on the tooth to be replaced and its position in the arch. All these digitally designed customized components are fabricated prior to surgery. This approach allows the transfer of the precise fit from the digital design and guided surgery not only to the implant but also to the prosthetic components, ensuring that the carefully planned treatment can be predictably replicated during the surgical–prosthetic phase [5,6,7,8].

The shape of the abutment can also influence peri-implant bone resorption. As shown in the study by Souza and colleagues, narrow and straight healing abutments lead to less bone remodeling than wide and divergent abutments [39]. The customized definitive FR abutment, which remains firmly connected to the implant after placement, mimics the shape of the cement–enamel junction (CEJ) of the tooth to be replaced, with the customized coping and provisional restoration designed directly on the abutment. The FR abutment performs a crucial role in protecting, shaping, and guiding the differentiation of the clot within the connective tissue. Simultaneously, the FR coping specifically directs the differentiation of the clot within the junctional epithelium, aided by the provisional restoration, in shaping the most coronal aspect of the osseomucosal seal. This approach facilitates immediate tissue conditioning, as the definitive abutment and other prosthetic components are optimally shaped and positioned during surgery and remain continuously connected thereafter [9,12,27,32,35,36]. In the described case, clinical assessments at 6-month and 1-year follow-ups demonstrated stable and healthy peri-implant tissue conditions.

Periapical radiographs confirmed the accurate positioning of the implant relative to the mesial tooth, the distal implant, and along the equicrestal apico-coronal axis. Therefore, as observed from the comparison of the three periapical radiographs taken at T1, T2, and T3, the mesial and distal bone peak levels exhibited minimal variation and remained coronal to the implant platform. By utilizing the definitive abutment, disconnections and reconnections as well as apical displacement of the connective tissue were avoided, maintaining a clinical condition of healthy peri-implant tissues and correct three-dimensional implant positioning [9,12,13,14,15,16,17,18,32].

Unlike the one-time abutments currently reported in the literature, based on the best available scientific evidence, the FR abutment, besides being definitive and remaining connected post-placement, is customized in mesial, distal, buccal, and lingual dimensions; height; and inclinations according to the tooth to be replaced and the dental arch involved. It integrates seamlessly into a digital implant–prosthetic workflow through patented digital mathematics. The customization of the abutment allows the peri-implant tissues to be conditioned as soon as the first millimeters of the peri-implant seal, starting as early as the time of implant placement, thus avoiding the delays and disconnections associated with indirect customization. The mucosal seal in its most coronal portion is customized by the FR coping, which is site-specific and varies depending on the tooth being replaced and its location within the dental arch [27,35,36,38]. The pre-surgically adequate keratinized mucosa width is preserved and matures over time. Supporting this approach, soft tissue volume analysis performed on the STL files at T0, T2, and T3 demonstrated a stable and increasing trend over time. This highlights the beneficial effect of placing a customized definitive abutment, customized coping, and provisional restoration in a single surgical–prosthetic session, promoting peri-implant tissue health and serving as a rigid scaffold for the clot.

Regarding the implant–abutment connection, numerous studies in the literature highlight the internal hexagonal connection as advantageous in terms of mechanical stability, reduction in tensile forces, effective seal against microleakage, precision, and resistance to high masticatory loads, ultimately leading to the preservation of peri-implant bone [40,41,42,43]. In this case report, an implant with an internal hexagonal connection was employed to precisely adapt the FR abutment’s hexagonal connection to the implant, aligning the vestibular side of the abutment’s hexagon with one side of the implant’s hexagon, as per the digital plan. At the 1-year follow-up, peri-implant marginal bone levels remained stable, likely due to the use of the FR abutment and other customized prosthetic components, which from the outset preserved and guided the clot. The customization of FR abutments concerns the portion above the abutment–implant connection. The abutment-to-implant connection varies based on the selected implant, according to the specific clinical needs and preferences of the clinician.

The FR technique is compatible with commonly utilized implant systems and can be applied to implant types I (implant placement immediately after tooth extraction and as part of the same surgical procedure), II (implant placement at complete soft tissue coverage of the alveolus, typically 4 to 8 weeks), III (implant placement at substantial clinical and/or radiographic bone filling of the alveolus, typically 12 to 16 weeks), and IV (implant placement at healed sites, typically more than 16 weeks). It requires proper digital planning of the implant and prosthetic FR components and saves time for the patient and dentist while respecting natural biological healing times and avoiding intermediate prosthetic components and additional impressions [12,20,32].

## 4. Clinical Relevance

The customized definitive FR abutment and associated prosthetic components integrated from the design stage into a complete digital workflow achieve predictable esthetic and biological peri-implant tissue stability, reducing time requirements for both the patient and the dentist. The FR abutment respects the connective tissue of the peri-implant seal because it is not subjected to further disconnections. Along with the FR coping, these components, customized for each tooth and arch, individualize the morphology of the peri-implant soft tissues from the moment of insertion. Although this case report offers meaningful preliminary insights, it is subject to inherent limitations. Most notably, the lack of a control group restricts the ability to generalize the outcomes to a broader population. Furthermore, as the analysis is based on a single clinical case, the findings should be interpreted within the context of these inherent constraints. These considerations highlight the need for further research, preferably through studies with larger cohorts and randomized controlled trial designs, to validate and expand upon the implications suggested by this technique.

## 5. Patents

Digital Mathematics of First Biological Respect “FR” components are the property of RMS SRL.

## Data Availability

The original contributions presented in this study are included in the article. Further inquiries can be directed to the corresponding author.

## Abbreviations

FR stands for ‘First Biological Respect’ and refers to a complete biodigital method used to virtually plan and execute not only the three-dimensional placement of the implant but also the customized FR definitive abutment, tailored to the specific tooth to be replaced and its location in the dental arch. The FR abutment, positioned during the surgical phase, protects and guides the clot during its differentiation. The FR abutment supports the FR customized coping and provisional and definitive framework, all of which are digitally designed and produced before the time of surgery, allowing for a full biodigital restoration technique. In order to incorporate the customized FR abutment and coping into the design, the digital mathematics of these components have been patented.
